# Main histological parameters to be evaluated in an experimental model of myocardial infarct treated by stem cells on pigs

**DOI:** 10.7717/peerj.7160

**Published:** 2019-07-22

**Authors:** Soledad García Gómez-Heras, Carlota Largo, Jose Luis Larrea, Luz Vega-Clemente, Miguel Calderón Flores, Daniel Ruiz-Pérez, Damián García-Olmo, Mariano García-Arranz

**Affiliations:** 1 Human Histology and Pathology, Universidad Rey Juan Carlos, Alcorcón, Madrid, Spain; 2 Experimental Surgery, La Paz University Hospital, IdiPaz, Madrid, Spain; 3 Surgical Cardiology Department, La Paz University Hospital, Madrid, Spain; 4 Cell Therapy laboratory, Health Research Institute, Fundación Jiménez Diaz, Madrid, Spain

**Keywords:** Pig, Myocardical infarction, Histological parameters, ASC

## Abstract

Myocardial infarction has been carefully studied in numerous experimental models. Most of these models are based on electrophysiological and functional data, and pay less attention to histological discoveries. During the last decade, treatment using advanced therapies, mainly cell therapy, has prevailed from among all the options to be studied for treating myocardial infarction. In our study we wanted to show the fundamental histological parameters to be evaluated during the development of an infarction on an experimental model as well as treatment with mesenchymal stem cells derived from adipose tissue applied intra-lesionally. The fundamental parameters to study in infarcted tissue at the histological level are the cells involved in the inflammatory process (lymphocytes, macrophages and M2, neutrophils, mast cells and plasma cells), neovascularization processes (capillaries and arterioles) and cardiac cells (cardiomyocytes and Purkinje fibers). In our study, we used intramyocardial injection of mesenchymal stem cells into the myocardial infarction area 1 hour after arterial occlusion and allowed 1 month of evolution before analyzing the modifications on the normal tissue inflammatory infiltrate. Acute inflammation was shortened, leading to chronic inflammation with abundant plasma cells and mast cells and complete disappearance of neutrophils. Another benefit was an increase in the number of vessels formed. Cardiomyocytes and Purkinje fibers were better conserved, both from a structural and metabolic point of view, possibly leading to reduced morbidity in the long term. With this study we present the main histological aspects to be evaluated in future assays, complementing or explaining the electrophysiological and functional findings.

## Introduction

During the last decade, many attempts have been made to develop a treatment for heart failure using experimental models of myocardial infarction (MI). Most of the attempts have been based on advanced therapies. Although promising advances have been achieved, trial results have limitations in the clinical phase (Chen et al., 2014; Kim et al., 2017; Liu et al., 2016).

The attempts that have reached clinical trial phases have used mesenchymal stem cells (MSCs). From diverse origins, MSCs are immunoprivileged cells that are suitable for allogeneic and xenogeneic transplantation (Aggarwal & Pittenger, 2005). As immunomodulatory cells, they act on the injured or inflamed area by secreting various growth factors. To do so, they stimulate the proliferation of local cells which also helps with remodeling the local matrix. In summary, they improve both healing and repairing of post-MI tissue (Wu et al., 2007). Currently, MSC-based therapies present a promising treatment plan for decreasing morbidity and mortality from chronic diseases with poor wound healing (García-Gómez et al., 2010).

To design new therapeutic approaches based on experience to date, we should consider two important factors: selecting the correct experimental model and choosing the most appropriate parameters to analyze.

Experimental models must meet minimum requirements, including being feasible and reproducing human disease as closely as possible. A porcine model has proven to be the most effective model for studying ischemic heart disease, due to the anatomical, physiological and pathological similarities between human and pig hearts (Dixon & Spinale, 2009). Most of these models are based on surgical techniques for coronary artery occlusion after thoracotomy (Hoffmann et al., 2004; Klocke et al., 2007; Litvak, Siderides & Vineberg, 1957) or are based on less invasive techniques (Krombach et al., 2005; Yoshimizu et al., 1986) that reproduce the ischemic lesion as accurately as possible. The most frequently used model involves anterior descending coronary artery occlusion (due to its simple and reproducible approach) with occlusions at various levels and a selective venous return. In all cases, an arterial occlusion lasting more than 1 h causes an irreversible effect similar to a permanent occlusion (Verdouw et al., 1998).

Considering the parameters to be analyzed, most studies have focused mainly on the acute phase of the infarction, and results have been based on clinical and electrophysiological parameters (Chen et al., 2014; Kim et al., 2017; Liu et al., 2016). In order to prevent and treat pathologies derived from the affectation of the coronary arteries and consequent cardiac failure, it is necessary to understand not only the clinical-electrophysiological parameters but also the pathophysiology of the disease and the progress of the tissues involved in MI.

Different types of cells have been used in experimental assays on pigs (Castro et al., 2019; Johnson & Singla, 2017; Mohsin & Houser, 2019; Shah et al., 2018; Van der Spoel et al., 2011; Wang et al., 2017). The types of cells that have been tested in various clinical trials (http://www.clinicaltrials.gov) with disappointing results have included bone marrow mononuclear cells (BMNCs), bone marrow mesenchymal stem cells(BMSCs), mesenchymal cells derived from adipose tissue (ASCs) and, more recently, pre-derived embryonic cells (ESCs), induced pluripotent stem cells (iPSCs) and cardiac progenitor cells (CSCs) (Higuchi et al., 2017).

We used a model of descending coronary artery occlusion to describe the pathophysiology of the process and the intracardiac injection of adipose-derived mesenchymal stem cells (ASCs), a type of MSCs that are abundant and easy to obtain, to analyze histological changes in both the infarcted tissue and the adjacent area. In this way, we were able to observe the effects of the intralesional application of ASCs.

Our research involved analyzing the changes that occurred during the inflammatory process, cicatrization and neovascularization, all of which are characteristic events in post-MI tissue regeneration. In addition, we studied the state of conservation of cardiac cells, cardiomyocytes and Purkinje fibers close to the infarcted region. To that end, we created an MI occluding the left anterior descending coronary artery and we compared the experimental treatment (intralesional injection of 5 × 10^6^ cells, MI + ASC group) with the natural evolution of infarcted tissue (saline injection, Myocardial Infarction Control (MIC) group). All the animals were euthanized after 4 weeks. Our objective was to determine, from a histopathological point of view, the effect of ASCs on the previously discussed patterns that are fundamental to understanding the functional consequences of long-term post-MI.

With this study we explored the main histological aspects to be evaluated in assays in MI animal models treated by stem cells. These studies can add rationality to understanding the electrophysiological and functional findings.

## Materials and Methods

### Animals

Nine Landrace-Large White pigs 23–28 kg were used: one was male -used to obtain ASCs from subcutaneous adipose tissue- and the rest were females destined for the intervention. All procedures were performed in the Experimental Surgery Department of La Paz University Hospital in Madrid, Spain. We followed the protocol approved by the Animal Welfare Ethics Committee (CEBA 14-2011) and complied with the EU Directive on experimental animals (63/2010 EU) and related Spanish legislation (RD 53/2013).

### Surgery

Twenty-four hours before surgery, the animals were pre-medicated with a fentanyl patch, and were administered 12 mg/kg ketamine (im), midazolam 0.5 mg/kg (im), and tramadol 5 mg/kg (im) 15 min before the intervention. The animals were anesthetized using isoflurane 2% and a continuous infusion of morphine (12 mg), ketamine (30 mg) and lidocaine (15 mg) in 500 ml saline solution at a rate of 10 ml/kg/h throughout the intervention.

A lateral thoracotomy was performed, and the anterior descending artery was ligated by 6/0 silk suture (Ethicon). One hour after ligation, 5 × 10^6^ stem cells or saline solution (1 ml) were delivered by syringe into the myocardial ischemic tissue around the infarct area (at 3 points). After surgery, the animals were kept individually isolated in a 2 m^2^ space with controlled food and water ad libitum. Analgesia was provided with a fentanyl patch every 2 days for a week, and the animals tolerated food the day after surgery. Ceftriaxone 40 mg/kg (im) was used as antibiotic prophylaxis from 3 days before until 72 h after the intervention.

### Isolation of Adipose-Derived Mesenchymal Stem Cells

Adipose tissue-derived stem cells (ASCs) were obtained from an animal of the same breed according to a previously described protocol with minor modifications (Zuk et al., 2001). After that, the cells were expanded in culture with Dulbecco’s Modified Eagle’s Medium, supplemented with 10% fetal bovine serum and 1% penicillin/streptomycin at 37 °C and 5% CO_2_. The cells were characterized by flowing cytometry and differentiated as adipocytes, osteocytes and chondrocytes, confirming that we were working with mesenchymal stem cells according to International Federation for Adipose Therapeutics criteria (Bourin et al., 2013). Finally, the cells were expanded in vitro and aliquots of 5 million were frozen with 10% dimethyl sulfoxide and stored in liquid nitrogen after use.

One week before the intervention, the required aliquots were defrosted and cultured until a sufficient number was obtained (5 × 10^6^ cells/animal). Before use, the cells were detached from the culture with trypsin-EDTA and were washed three times with phosphate buffered saline (PBS, Gibco).

Prior to injection, they were marked with CelltrackerDil (Invitrogen, Carlsbad, CA, USA) according to the manufacturer’s instructions in order to identify ASCs in animal tissue samples. The cells were located in red spectra (553/570 nm) by fluorescence microscopy.

### Obtaining Samples

In all cases, a 2 mL sample of blood was drawn prior to surgery and 24, 48 and 72 h post-surgery, for the purpose of analyzing troponin and biochemical parameters.

All animals were euthanized 1 month after surgery by intravenous injection of 5M potassium chloride, having previously been anesthetized using isoflurane 2%. The hearts were extracted and washed with 10% formaldehyde through the coronary artery and mitral valve. Once the tissues were fixed, samples were obtained from the infarct area and periphery.

### Histological analysis

Five mm^3^ samples were fixed in 10% formaldehyde at room temperature, embedded in paraffin and cut into 5-micron-thick slices in a Micron HM360 microtome.

Sections were stained with hematoxylin-eosin to identify plasma cells, lymphocytes, neutrophils and capillaries, using toluidine blue for the identification of mast cells.

For the immunohistochemical studies, histology sections were deparaffinized and rehydrated before endogenous peroxidase activity was blocked with H_2_O_2_ (0.3%) in methanol. The slides were rinsed with PBS and incubated with primary antibodies in a moist chamber at room temperature. The sections were subsequently incubated with biotinylated anti-rabbit IgG and LBA (DAKO) for 25 min at room temperature, rinsed with PBS and immersed for 25 min in avidin peroxidase. The immunostaining reaction product was developed using diaminobenzidine. Counter staining was performed with hematoxylin. The specificity of the immunohistochemical procedure was confirmed by incubation of sections with non-immune serum instead of a primary antibody.

The primary antibodies used were anti-CD14 antibody (MyBioSource, MBS-2027456, 1/500), anti-CD163 antibody (Serotec, MCA242GA, 1/200), muscle specific actin monoclonal antibody (Novocastra, A7811, 1/100), Desmin Monoclonal Antibody (Novocastra, DE-R11, 1/50), Connexin 43 mouse monoclonal antibody (Cell Signaling Technology, cst-3512, 1/50), and HIF1-α antibody (Gene Tex, GTX 30105, 1/1000).

The Masson trichrome technique was used to evaluate the degree of fibrosis (percentage of collagen area [blue] versus tissue area [red]).

All histological slides were studied under a Zeiss Axiophot 2 microscope and photographed with an AxiocamHRc camera. Twenty contiguous non-overlapping fields (200 × or 400 ×) per slide from each group were counted according to the Novotny et al. (2018) and the Yuetal2018 protocols. All the cells were quantified by the same researcher without knowledge of the groupings.

### Statistical analysis

Heart rate differences between groups were assessed using the corrected chi-squared test. Results with a *p* value of less than 0.05 were considered significant.

## Results

### Animal model

The experimental animal model we created showed high infarction reproducibility due to occlusion of the left anterior descending coronary artery in its upper third, as well as a high animal survival rate (90%). In all cases, the necrotic tissue was in the same anatomical region, was approximately 1.4 cm (±0.2) in diameter, and was associated with ST elevation as shown in the electrocardiogram. Subsequently, MI was confirmed by a >20% increase in basal troponin levels at 24 h post occlusion and a decrease of those levels by about 50% at 48 h.

### ASC location after injection

**Figure 1:**
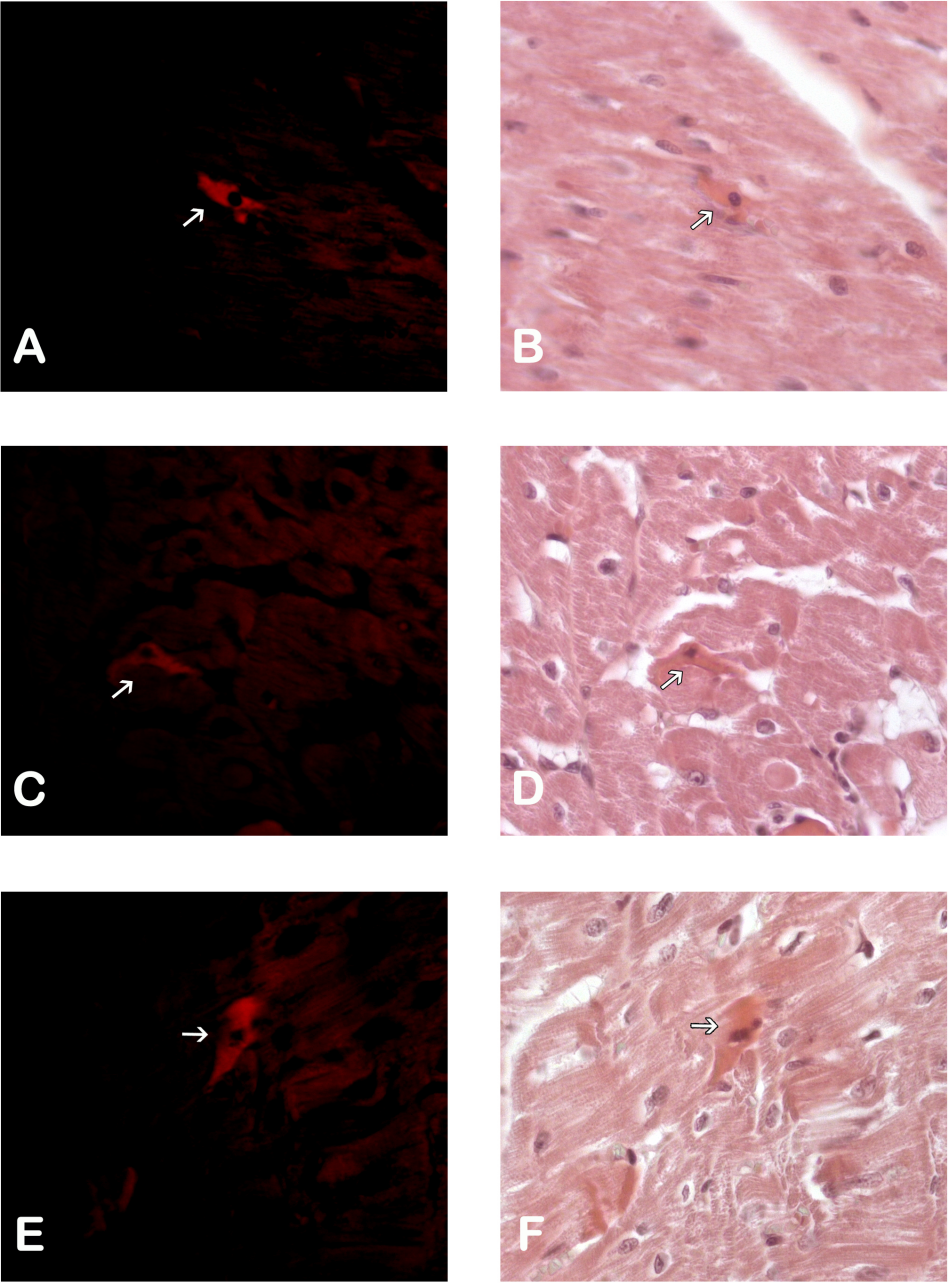
Adipose Stem Cells in the myocardium of MI+ASC heart group. (A, C and E) Fluorescence Microscopy Celltracker Dil 400 ×; (B, D, and F) Hematoxylin-eosin, 400 ×.

Immunohistological detection of the DiL signal in the cicatricial region was observed in the IM + ASC group after one month. As expected, no positive DiL signal was observed in the control hearts (Fig. 1).

### Histological results (Tables 1 and 2)

#### Inflammatory reaction

After the quantitative analysis of the infarcted area, we observed a greater infiltration of plasma cells and mast cells (2:1) in the animals treated with ASCs (MI + ASCs). In addition, the quantity of the neutrophils was less (1:3) and the number of lymphocytes was similar in both groups.

**Table 1:** Histological results in the scar area.

SCAR AREA		
	MI+ASCs	MIC
PLASMA CELLS 20X* ($\bar {x} \pm $se)	8.73 ± 0.50	3.7 ± 0.41
NEUTROPHILS 40X* ($\bar {x} \pm $se)	9.05 ± 0.68	12.86 ± 0.74
LYMPHOCYTES 40X ($\bar {x} \pm $se)	8.46 ± 0.75	7.06 ± 0.55
MAST CELLS 20X* ($\bar {x} \pm $se)	1.34 ± 0.17	0.51 ± 0.11
MACROPHAGES 20x ($\bar {x} \pm $se)	13.97 ± 0.94	15.12 ± 0.81
MACROPHAGES M2 20X* ($\bar {x} \pm $se)	2.65 ± 0.38	0.75 ± 0.13
CAPILLARIES ($\bar {x} \pm $se)	9.02 ± 0.57	9.38 ± 0.64
ARTERIOLES ($\bar {x} \pm $se)	3.13 ± 0.23	2.31 ± 0.19
SCAR AREA (µm^2^)	26.15 ± 1.96	23.18 ± 2.02
SCAR FIBROSIS	DENSER	LAX

**Notes.**

* *P* < 0.05.

se standard error $\bar {x}$ average

**Table 2:** Histological condiction of cardiac cells in the pericicatricial zone.

**PERICICATRICIAL ZONE**				
	**CARDIOMYOCYTES**		**PURKINJE FIBERS**	
	MI+ASCs	MIC	MI+ASCs	MIC
LOSS OF THE ACTINE PATTERN	+	++	++	+++
LOSS OF THE DESMIN PATTERN	+	++	+	++
CHANGES IN GAP JUNCTION	+	++	+	++
NUCLEOUS POSITIVEFOR ANTI- HIF-1α ANTIBODY*(% positive nuclei)	34.75(45.006%)	13.75(18.1%)	5.63 (56.44%)	4.32(23.64%)

**Notes.**

- NONE + MILD ++ MODERATE +++ SEVERE

* *P* < 0.05.

**Figure 2:**
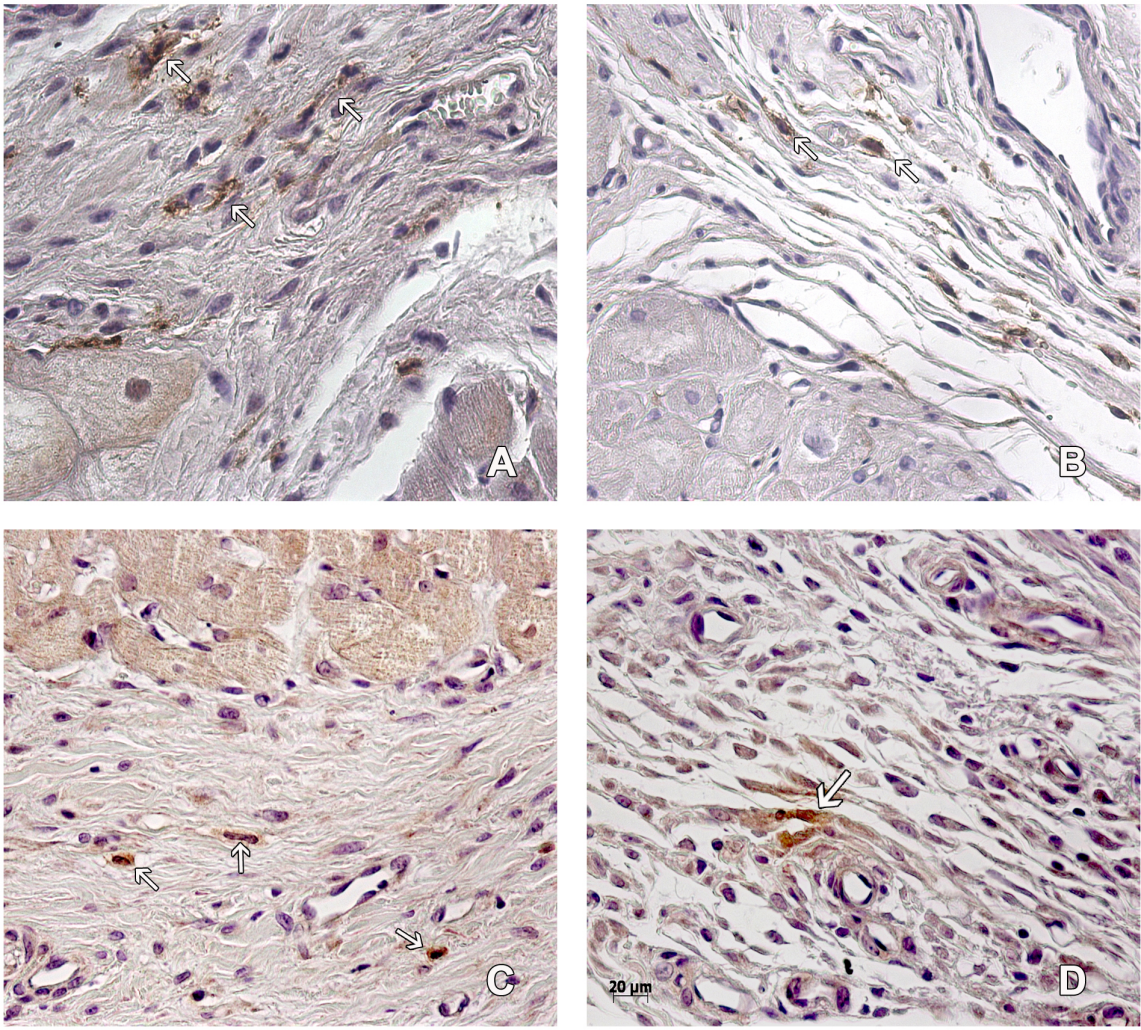
Macrophages in the peri-cicatricial area. The number of macrophages (arrow) in the peri-cicatricial area of MI+ASC heart (A) is similar to MIC group (B). Immunohistochemistry anti-CD14, PAP. 400 ×. M2 macrophages (arrows) are in greater proportion in MI+ASC group (C) than in MIC (D). Immunohistochemistry anti-CD163 PAP. 400 ×.

The amount of CD14+ macrophages quantified in the infarcted area was greater in the MIC group. CD163+ macrophages were present in greater amounts in the MI + ASC group (15.17% of CD14+ macrophages were CD163+). In the MIC group, 4.95% of the CD14+ macrophages were CD163+ (Fig. 2).

We did not find inflammatory infiltrate outside the infarct area in any of the animal groups studied.

#### Vascular density

**Figure 3:**
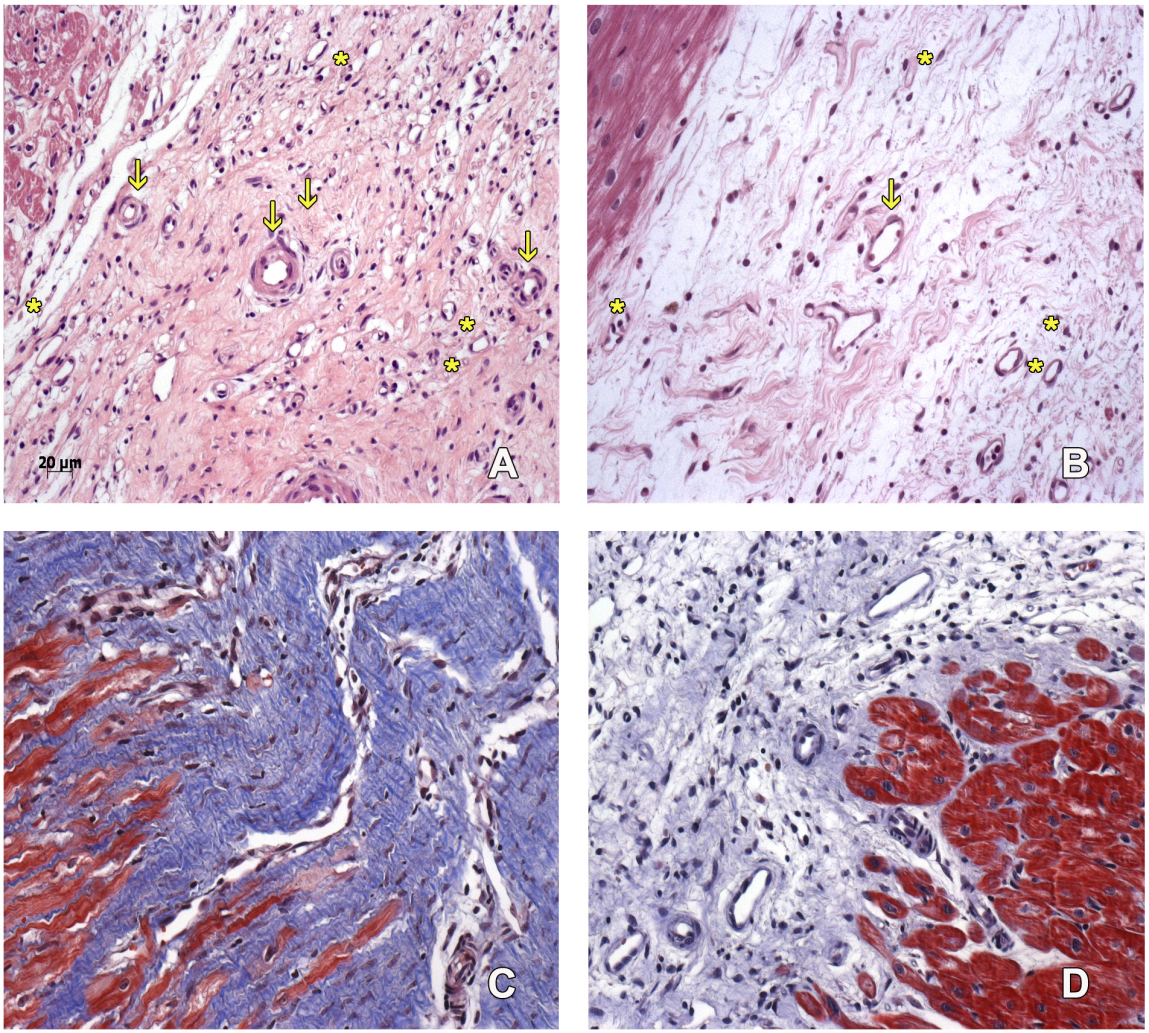
Scarring region. Capillaries (asterisk) and arterioles (arrows) in the scarring region of MI+ ASC hearts (A) and MIC hearts (B). Hematoxylin-eosin, 200 ×. Denser scar in ASC group (C) than in Control group (D). Masson, 200 ×.

The number of capillaries was similar in both groups. We found more arterioles in the scarring region of the hearts belonging to the MI + ASC group than in the MIC group (at a ratio of 3:2) (Figs. 3A–3B).

#### Collagen deposition

A denser and more organized scar was found in the MI + ASC group than in the MIC group. In both groups, fibrosis extension was similar (Figs. 3C–3D).

#### Cardiomyocytes in the peri-cicatricial region

**Figure 4:**
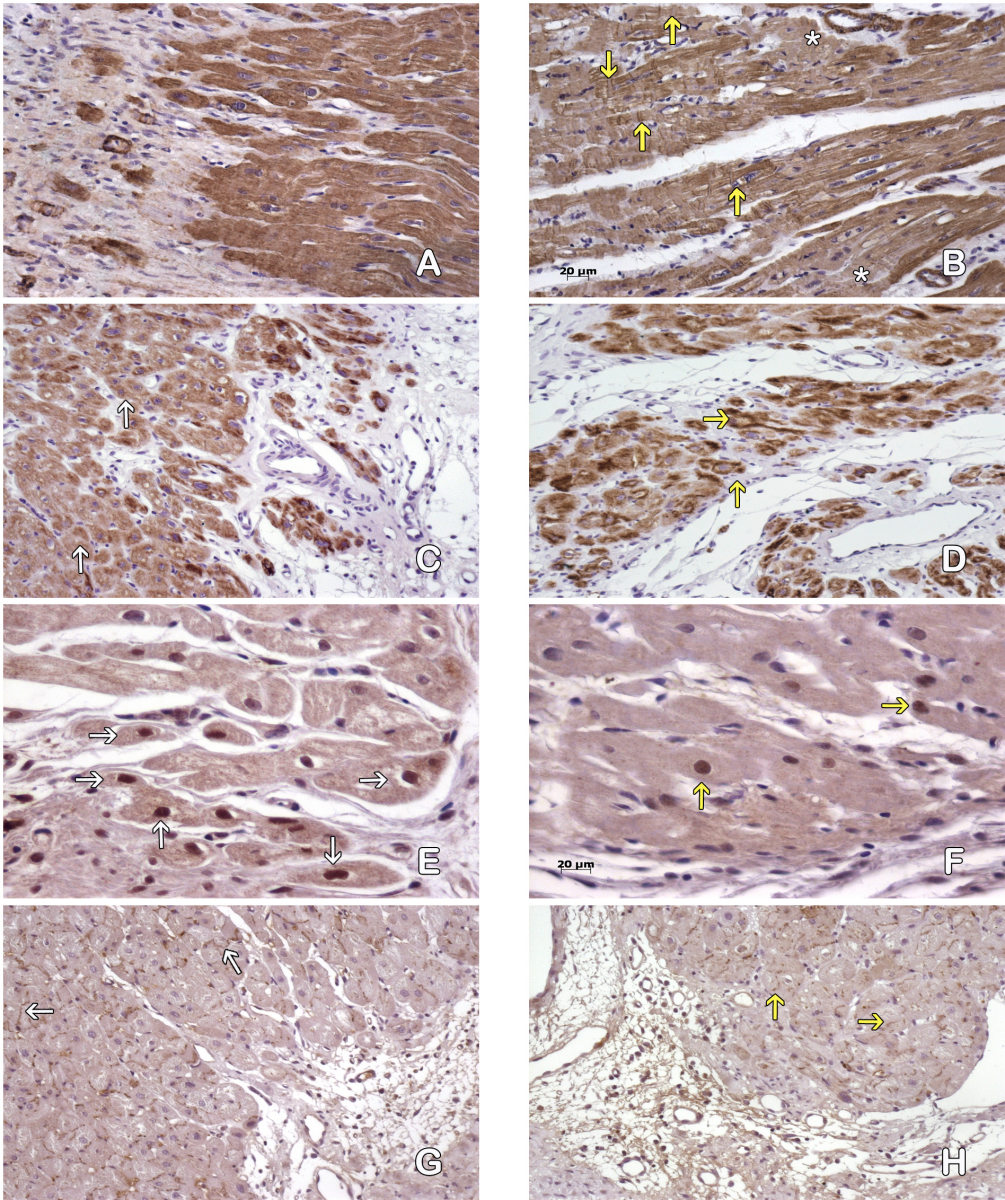
Cardiomyocytes in the peri-cicatricial zone. Labeling of actin demostrate little areas with loss of actine expression in MI+ASC hearts (A), in MIC group (B) actin pattern is lost in bigger zones (asterisk). We can see cells with contraction-bands (yellow arrows). Immunohistochemistry anti-actin, PAP. 200 × Desmin expression is better conserved (white arrows) in MI+ASC group (C) than MIC group (D, yellow arrows). Immunohistochemistry anti-desmin, PAP. 200 ×. Greater number of HIF-1α-positive nuclei (white arrows) in MI+ASC (E) than in MIC group (F, yellow arrows). Immunohistochemistry anti- HIF1-α, PAP. 400 ×. Connexin 43 is better conserved in MI+ASC hearts (G, white arrows) than in MIC hearts (H) where it is diminished and granular (yellow arrows). Immunohistochemistry anti connexin 43, PAP. 200 ×.

The MI + ASC group had better conservation of the cytoskeleton than the MIC group based on actin/desmin staining (Figs. 4A–4D).

Studying the distribution of Connexin 43 cells, we observed that, in the MI + ASC group, there was protein expression, and its distribution was conserved. In the MIC group, the expression of Connexin 43 was reduced and its distribution was altered (Figs. 4E–4F).

Immunohistochemistry revealed HIF1-α protein throughout areas of perinfarcted myocardium. In the MI + ASC group, 45% of the cardiomyocytes had positive nuclei for HIF-1α, whereas in the MIC group, only 18.1% of them were positive (*p* < 0.05) (Figs. 4G–4H).

#### Purkinje fibers in the peri-cicatricial region

**Figure 5:**
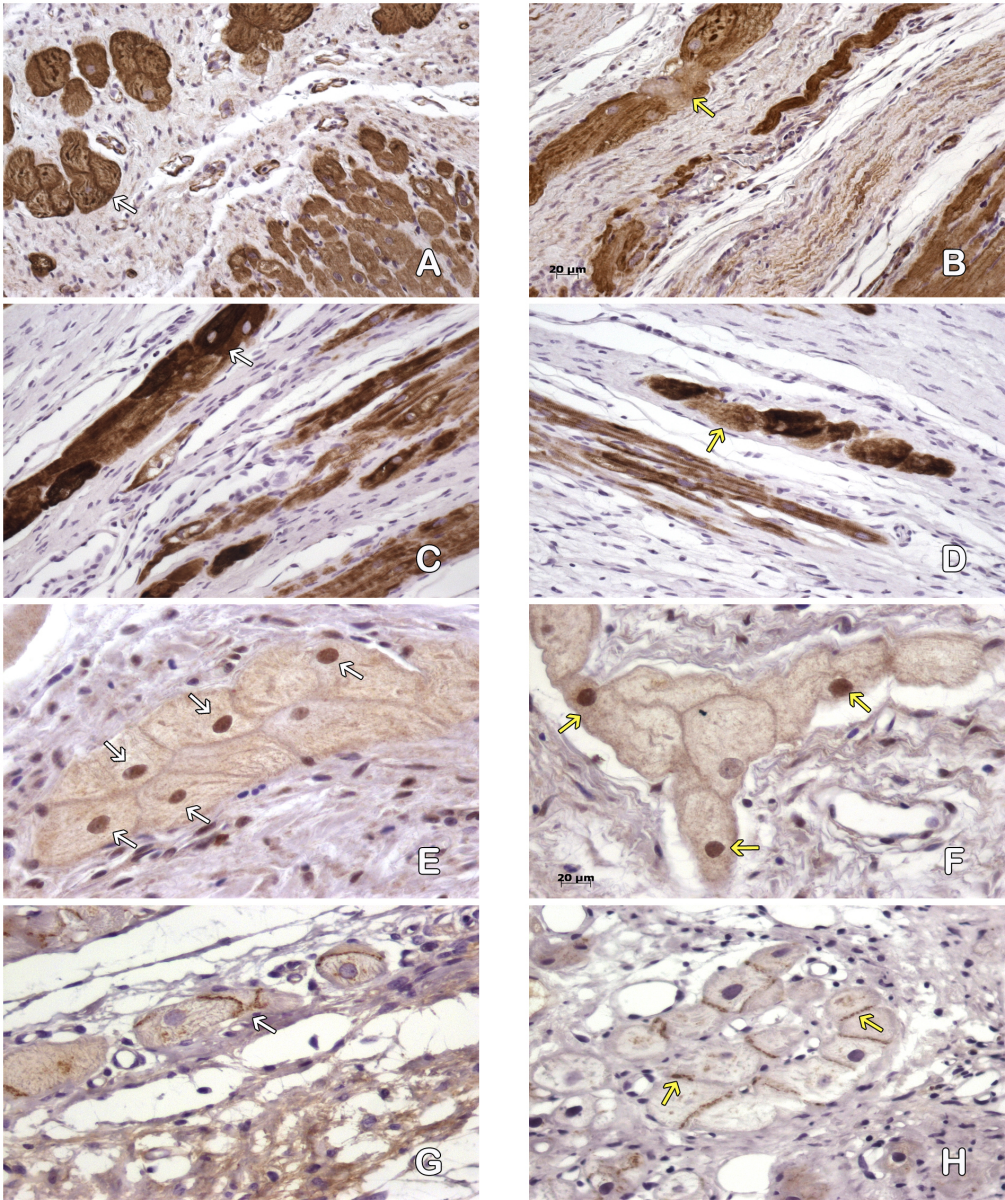
Purkinje fibres in the peri-cicatricial zone. Actine filaments in the Purkinje fibers are better conserved in MI+ASC (A, white arrows) than in MIC ones (B, yellow arrows). Immunohistochemistry anti-actin, PAP. 200 ×. Most of the desmine filaments are better conserved in MI+ASC group (C, white arrows) than in the MIC group hearts (D, yellow arrows). Immunohistochemistry anti-desmin, PAP. 200 ×. More positive nuclei (white arrows) in the cells of pericicatricial zone in MI+ASC group (E) than in control group (F, yellow arrows). Immunohistochemistry anti- HIF1-α, PAP. 400 ×. Better conserved pattern of connexin 43 between Purkinje fibers in MI+ASC group (G, white arrows) than in control group where, also, it is diminished (H, yellow arrow). Immunohistochemistry anti-connexin 43, PAP. 400 ×.

The structure of the cytoskeleton of the MI + ASC group was well preserved, whereas in the MIC group we observed a decrease of filaments (Figs. 5A–5D).

The immunohistochemical technique reflected a decrease in the expression of Connexin 43 cells in the gap junctions of the Purkinje fibers in the MI + ASC group, a decrease that was even more marked in the MIC group, in which its distribution was irregular (Figs. 5E–5F).

Based on the expression of HIF-1α, immunoreactivity was observed in the nuclei of the Purkinje fibers throughout the areas of perinfarcted myocardium and was not present in non-infarcted myocardium. In the MI + ASC group, 56.44% of the Purkinje fibers had positive nuclei, whereas in the MIC group, only 23.64% had positive HIF-1α nuclei (*p* < 0.05) (Figs. 5G–5H).

## Discussion

Various studies have been published on myocardial infarction models after a permanent ligation, especially in the acute phase. Most of these have focused on evaluating the functionality and electrophysiology of the proposed treatments (De Siena et al., 2010; Kim et al., 2017).

In our study, we emphasized the pathological aspects more than the electrophysiological ones. At the electrophysiological level, we observed a decrease in systolic and diastolic pressure after the infarction which, although it did not normalize completely, improved hours after the infarction. Although this normalization was not significant, it was observed earlier in the animals treated with cells. Also, we observed a decrease in heart rate in animals treated with cells less than one hour post-cell injection, which implies a lower cardiac output and an improvement in the state of the animals. At the blood protein level, the most significant parameter was troponin, and we observed post-infarct that troponin levels decreased at 48 h more than 50% in animals treated with cells, while the control group did not reach a 50% decrease in that period of time. These better data in the group treated with cells coincides with the pathophysiological data described: a lower inflammatory infiltrate, less fibrosis and a better conservation of the parenchyma.

For our study, we analyzed the three main factors that could lead to an increase in post-infarction morbidity: a chronic inflammatory process, scarring characteristics and the status of myocardial cells remaining around the infarcted area, at a time specific to its evolution and comparing a normal evolution with experimental cell therapy treatment.

One of the main goals in regenerative medicine and tissue engineering in the MI field is initial inflammatory response modulation. As Kocher et al. (2001) had already reported on the anti-inflammatory effect of Mesenchymal Stem Cells (MSCs) in myocardial infarct. Since then, numerous research groups have published articles referring to multiple reasons for the infusion of MSC to shorten and regulate the inflammatory process, for example the regulating T or T-native cells, the M1/M2 macrophage transition, the secretion of interleukins like IL10 or IL-4. (Najar et al., 2016; Krampera et al., 2003; Yañez et al., 2010; Hirose et al., 2018). Actually, many studies have been carried out and they all have found different reasons to explain this shortening of the acute inflammatory process, but there may be many other aspects that generate this effect as suggested in 2012 by Georgiev-Hristov et al. (2012).

The principal problem is a prolongation of the inflammatory phase during the wound healing process leading to adverse scarring and causing medium/long term cardiac failure (Chen & Frangogiannis, 2016; Leor et al., 2016). Along these lines, it is necessary to analyze the qualitative histological characteristics of that reaction: it should attract reparative cells, such as M2 macrophages, which favor the formation of well vascularized tissue, and with the right proportion of collagen for proper ventricular function.

Our results show that plasma cells decrease in the infarcted tissue in an untreated heart. However, they were attracted to the inflammatory-reparative area by the ASCs and they infiltrated the infarcted tissue of the hearts treated with ASCs. Similar to other authors (Mazo et al., 2012; Rasmusson et al., 2005; Rasmusson et al., 2007), we consider that this increase in plasma cells in the treated animals might be due to the paracrine activity of mesenchymal cells.

When we analyzed the various cells involved in the inflammatory process, we found that the control group maintained a high number of neutrophils and an increase in macrophages, which reveals an acute inflammation or an initial phase of a chronic inflammatory stage. On the other hand, ASC treatment caused late chronic phase development of a decrease in neutrophil number and an increase in type 2 macrophages. This outcome might be due to the anti-inflammatory effect of ASCs (García-Gómez et al., 2010; Kuo et al., 2012; Van den Akker, De Jager & Sluijter, 2013) and the shortening of the inflammatory-reparative process generated by the ASCs.

Another interesting result that we observed was that mast cell infiltration in the infarcted area was three times higher in the ASC-treated animals. We believe the lack of statistical significance between both groups was due to the fact that this infiltration by mast cells in an MI persists during the inflammatory process from the chronic stage (Levick et al., 2011; Reid et al., 2011). The mast cells seem to be involved in the paracrine regulation of growth factors (GF) in the infarcted area, although their exact functions must be further studied (Gentek & Hoeffel, 2017).

Macrophages change their phenotype and function in response to signals from the microenvironment. The M1/M2 balance may influence cardiac repair improvement and post-MI function (Leor et al., 2016; Gombozhapova et al., 2017). Therefore, this approach could be used as a therapeutic tool. Interactions between ASCs and macrophages are known: ASCs increase the expression of the M2 phenotype (Ryabov et al., 2018). In post-MI healing, a prolonged presence of M1 macrophages can lead to an increase in the size of the MI area and prevent correct resolution. We observed this increase of the MI area in our control animals. On the other hand, in treated animals we observed an increase in M2 macrophages to implicate a diminution in MI area. Our results, in accordance with previous data, showed a better condition of the infarcted tissue in the IM-ASC group animals.

Also, ASCs present angiogenic effects when implanted in infarcted tissue. Numerous published studies have demonstrated this approach (Chou et al., 2014; Citro et al., 2014; Kim et al., 2014). In our study, after 1 month of evolution of the ASC group, we did not observe an increase in capillaries. Although we detected a greater number of arterioles, the difference was not statistically significant. We believe that 1 month of evolution of the infarction led to stabilization of the angiogenesis of the infarcted region. A study with analysis of shorter periods could possibly clarify this difference.

In our study we observed a dense scar associated with better organization of collagen type I in the ASC group, that possibly explained the better remodeling of the perinfarcted tissue and the generation of a more organized scar. Results are in accordance with those observed by other authors (Nong et al., 2011; Yu et al., 2018a; Yu et al., 2018b).

Free radical cardiac myoglobin and other sources play an important role in myocardial infarction (Zhu & Zuo, 2013). These oxygen radicals can potentially influence cardiac inflammation and the survival rates of injected stem cells. Thus, further studies into the correlation between reactive oxygen species and inflammation should be carried out in the future.

Finally, we determined the survival conditions of cardiac cells, cardiomyocytes and Purkinje cells. Different studies had previously implicated both cell types in remodulation of infarct tissue and a positive response to MSC treatment (Li et al., 2010; Muguruma et al., 2006; Shafei et al., 2017; Yoon et al., 2005). In general, we can conclude that in the MI + ASC group after 4 weeks of post infarction evolution, the cardiomyocytes and Purkinje cells were better conserved, and we can affirm this point by Connexin 43 and HIF1-α status. From both qualitative and quantitative points of view, the cytoskeleton filaments of these cells and the gap junctions (Connexin 43) in ASC group were similar to non-pathological conditions. After tissue hypoxia, affected cardiac cells respond to various mechanisms aimed at restoring cellular oxygen levels. One of the main response pathways is the inhibition of hydroxylation of HIF1-α by prolyl hydroxylases (PHDs), which translocate to the nucleus, initiating the transcription of factors that support normoxia: promoting angiogenesis, increasing cell proliferation and migration, stimulating glycolysis, etc. With all this activity, cell survival is more likely (Szade et al., 2015). Thus, stabilization and accumulation of HIF1-α is considered cardioprotective, helping preserve myocardial structure and function (Cheng et al., 2016; Lee et al., 2000; Townley-Tilson, Pi & Xie, 2015; Wu et al., 2015). After 4 weeks of postinfarction evolution, we observed how the presence of ASCs coincided with a greater proportion of cardiomyocytes and Purkinje fibers expressing nuclear HIF1-α with respect to the control group. Therefore, in these ASC-treated hearts, there was a more intense adaptive response to hypoxia conditions. Given that HIF1- *α* is a mechanism which starts immediately after the decrease in oxygen levels, it was maintained 4 weeks after MI, especially in the MI-ASC group.

We have also demonstrated that the infarction model carried out by the occlusion of the anterior coronary artery was reproducible. One month of evolution represents a period that is similar to what we observe in the clinic.

In conclusion, in this study we have shown the main histological parameters to be assessed after the generation of an infarction: the cells involved in the inflammatory process, cicatricial and neovascularization processes characteristic of post-MI tissue regeneration as well as in the conservation of cardiac cells, cardiomyocytes and Purkinje fibers adjacent to the infarcted area. Finally we used Stem Cell treatment to demonstrate the implications of these histological parameters in infarct tissue remodulation.

Thus, in this study, we propose what we consider to be the main histological aspects to be evaluated in future assays, and provide complementary explanations for the electrophysiological and functional findings.

##  Supplemental Information

10.7717/peerj.7160/supp-1 Dataset S1 Raw Data Arterioles (200 × field)

10.7717/peerj.7160/supp-2 Dataset S2 Raw data Capillaries (200 ×)

10.7717/peerj.7160/supp-3 Dataset S3 Raw data CD14 (200 × field)

10.7717/peerj.7160/supp-4 Dataset S4 Raw Data CD163 (200 × field)

10.7717/peerj.7160/supp-5 Dataset S5 Raw Data HIF Cardiomyocytes (200 × field)

10.7717/peerj.7160/supp-6 Dataset S6 Raw Data HIF Purkinje (200 × field)

10.7717/peerj.7160/supp-7 Dataset S7 Raw Data Lymphocytes (400 ×)

10.7717/peerj.7160/supp-8 Dataset S8 Raw Data Mast Cells (200 × field)

10.7717/peerj.7160/supp-9 Dataset S9 Raw Data Neutrophils (400 ×)

10.7717/peerj.7160/supp-10 Dataset S10 Raw Data Scar área (200 × field)

10.7717/peerj.7160/supp-11 Dataset S11 Raw Data plasma cells (200 × field)

## Additional Information and Declarations

### Data Availability

The following information was supplied regarding data availability: The raw data are available in the Supplemental Files. All histological slides were studied under a Zeiss Axiophot 2 microscope and photographed with an Axiocam HRc camera. Twenty contiguous non overlapping fields (200 × or 400 ×) per slide from each group were counted. They are the number of each type of imflamatory cell, HIF1-alfa positive nuclei and fibrosis area in each field.
